# Medical Opinion and Movement

**Published:** 1907-10-05

**Authors:** 


### Medical Opinion and Movement.

Professor Bier's methods of treatment of local inflammatory conditions by the induction of passive hyperæmia has received so much attention of late that it is already well known to the profession. In the limbs passive congestion is produced by the application of an elastic bandage above the inflammatory area. On the trunk and head this method is not applicable, and Dr. Klapp, Professor Bier's assistant, has devised a method of producing hyperæmia in these parts by means of glasses, similar to cupping glasses, attached to small india-rubber bags. The glass is applied with the bag compressed, and a partial vacuum results. The part to which the glass is applied projects and becomes of a reddish-blue colour. This cupping method may also be applied to the limbs in conjunction with the elastic bandage. Some successful results of the treatment in cases of tuberculous sinuses due to bone disease have been recently published by Dr. J. W. Sever. The treatment was carried out for one hour daily. The cups were removed every few minutes to renew the vacuum, and at no time was the suction strong enough to cause pain.

Some interesting facts were brought out in the course of the proceedings at the annual dinners of the old students of the principal London hospitals, on October 1. Mr. F. S. Eve, referring to the statue of Queen Alexandra, by Mr. Wade, about to be erected in the grounds of the London Hospital, stated that it would probably be unveiled next spring. The London Hospital School had obtained the highest entry of medical students on record. It was incidentally mentioned by Mr. W. D. Hoare that the quinquennial appeal would be for the sum of £500,000. At St. Thomas's Hospital Dr. T. Dyke Acland was distinctly humorous. He referred to the medical enthusiast who has declared that a good time was coming when mankind would cease to take medicine. Dr. Acland dissented from this view on the ground that it exhibited a very considerable lack of knowledge of human nature. He believed that so long as man endured he would continue to consume medicine. He wisely dwelt on the imperative necessity which exists for the establishment of a proper teaching university in London. Mr. Wainwright mentioned that St. Thomas's Hospital requires £7,500 a year additional income from voluntary contributions. At St. Bartholomew's Hospital the dinner was held in the great hall of the institution and attracted over two hundred guests. Both Dr. Acland and Mr. Harrison Cripps declared against the State endowment of either charity or science. Mr. Harrison Cripps claimed for St. Bartholomew's Hospital the advantage as a teaching institution of obtaining under a single roof facilities for teaching the science, theory and practice of medicine. It is expected that the pathological block will be completed in July 1908.

Surgeons cannot be too insistent upon attention to small details in the care of patients before, during and after operation. Many of the most successful surgeons ascribe their favourable results to their methods of treatment apart from the technique of the operation itself. It is always instructive therefore to hear from individual surgeons what are these details of their particular methods of procedure. Mr. J. H. Dauber has recently expressed in The Hospital his views on the subject, and although he himself would doubtless hardly claim originality for most of them, many of his remarks are worth repeating and recording, especially for the benefit of those members of the profession who are not exclusively employed in the domain of surgery. A proper consideration of the patients' mental condition before operation is very important, but often neglected. They are in a state of nervous tension and highly impressionable. Every influence about them should therefore be optimistic. Preparations for operation should be kept from them, and if possible they should be anæsthetised before they are brought into the operating room. In regard to the operation, Mr. Dauber is, of course, emphatic about asepsis, and advocates india-rubber armlets as well as gloves.

One point we most heartily endorse, and would specially commend to the attention of many eminent surgeons of the present day. Mr. Dauber says: "If there is one thing I believe in it is rapidity in operating. It is customary to say, 'The rapid operating of Fergusson would be out of place to-day; with anæsthesia we can operate at our leisure.'" No greater fallacy was ever promulgated. It is a fallacy, however, which is largely practised, and is probably answerable for more failures than would be credited by many surgeons.

A little smart business capacity, a little originality in advertisement, and any amount of shameless humbug are the essentials to success in foisting a useless remedy as an infallible cure for any and every disease upon a credible and gullible public. As Mr. Henry Sewill said lately in an address on the subject, quackery at the present day provides the safest field for the operations of cynical knavery. As he points out, nothing is likely to be done so long as the present apathy prevails on all sides, and he appeals to the British Medical Association to make some organised effort to suppress this growing evil. The question is utterly ignored by the public and by both Houses of Parliament, and there is a certain prejudice against the profession which militates against the success of any measures for its protection. Finally, and perhaps most important of all, the lay press is so dependent upon the quack advertisers for financial support, that there is no shadow of a possibility that the cry of reform might be taken up by any of the journals. It is estimated that not less than £1,500,000 is spent yearly in quack advertisements. Mr. Sewill's panacea is a Royal Commission. What is most urgently needed is the enlightenment of public opinion, and we have reason to doubt whether a Royal Commission would accomplish this object. In any case, it is to be feared we are a long way yet from obtaining even a Royal Commission on the subject.

